# Antibiotic prescription errors: the relationship with clinical competence in junior medical residents

**DOI:** 10.1186/s12909-022-03499-0

**Published:** 2022-06-14

**Authors:** Joshua Martínez-Domínguez, Octavio Sierra-Martínez, Arturo Galindo-Fraga, Juan Andrés Trejo-Mejía, Melchor Sánchez-Mendiola, Eric Ochoa-Hein, Mirella Vázquez-Rivera, Carlos Gutiérrez-Cirlos, Jesús Naveja, Adrián Martínez-González

**Affiliations:** 1 Coordination of Educational Development and Curricular Innovation (CODEIC), National Autonomous University of Mexico (UNAM), Mexico City, México; 2 Faculty of Medicine, National Autonomous University of Mexico (UNAM), Mexico City, México; 3 Manuel Gea Gonzalez General Hospital, Mexico City, México; 4 Salvador Zubirán National Institute of Medical Sciences and Nutrition, Mexico City, México; 5 National Institute of Pediatrics, Mexico City, México; 6 Internal Medicine, Salvador Zubirán National Institute of Medical Sciences and Nutrition, Mexico City, México; 7 Internship and Social Service, Faculty of Medicine, National Autonomous University of Mexico (UNAM), Mexico City, México; 8 Chemistry Institute, National Autonomous University of Mexico (UNAM), Mexico City, México; 9 Public Health Department, Faculty of Medicine, National Autonomous University of Mexico (UNAM), Mexico City, México; 10 Consejos académicos, Circuito exterior, Ciudad Universitaria, C. P. 04510 Coyoacán Ciudad de México, México

**Keywords:** OSCE, Assessment, Ambulatory medicine, Antibiotics prescription, Infectious diseases

## Abstract

**Background:**

A large portion of prescribing errors can be attributed to deficiencies in medication knowledge. These errors are preventable and most often occur at the time of prescription. Antimicrobials are the drug class most common incorrectly prescribed.

**Objective:**

To characterize the relationship between clinical competence and antibiotic prescription errors. We also investigated the frequency and severity of antibiotic prescription errors to identify items and attributes of clinical competence which are correlated with the antibiotic prescription error ratio.

**Method:**

A cross-sectional study was applied to assess clinical competence of junior medical residents in two reference academic hospitals and a regional hospital in Mexico City. It was conducted during February 2019. We used an infectious disease Objective Structured Clinical Examination (OSCE) to assess clinical competence and a measure of frequency, and severity of antibiotic prescription errors.

**Results:**

The number of eligible participants was ~ 255 (hospital meeting attendance), and the number of residents in this study were 51 (~ 20%), 31 were female (60.8%). The mean OSCE score was 0.692 ± 0.073. The inter-item (Cronbach’s alpha = 0.927) and inter-station internal consistency was adequate (Cronbach’s alpha = 0.774). The G coefficient in generalizability theory analysis was 0.84. The antibiotic prescription error ratio was 45.1% ± 7%. The most frequent category of severity of antibiotic prescription errors was category E (errors that may contribute to or result in temporary harm to the patient and require intervention), 235 (65.2%). We observed a negative and significant correlation between clinical competence and antibiotic prescription errors (*r* = -0.33, *p* < 0.05, CI95% -0.57 to -0.07), which remained significant after controlling for the effect of gender and time since graduation from medical school (*r* = -0.39, *p* < 0.01, CI95% -0.625 to -0.118). Using exploratory factor analysis we identified two factors, which explained 69% of the variance in clinical competence, factor 1 evaluated socio-clinical skills and factor 2 evaluated diagnostic-therapeutic skills. Factor 2 was correlated with antibiotic prescription error ratio (*r* = -0.536, *p* < 0.001).

**Conclusions:**

We observed a negative correlation between clinical competence and antibiotic prescription error ratio in graduated physicians who have been accepted in a medical specialty. The therapeutic plan, which is a component of the clinical competence score, and the prescription skills had a negative correlation with antibiotic prescription errors. The most frequent errors in antibiotic prescriptions would require a second intervention.

**Supplementary Information:**

The online version contains supplementary material available at 10.1186/s12909-022-03499-0.

## Introduction

Medical prescribing errors may be defined based on causes, processes, and outcomes. One definition includes all of these: “an act of omission or commission in planning or execution that contributes or could contribute to an unintended result” [1]. Reason differentiates between slips or lapses and errors. A slip or lapse occurs when the action conducted is not what was intended, reflecting an error of execution. Furthermore, a slip considered to be is observable, whereas a lapse is not. For example, turning the wrong knob on a piece of equipment would be a slip; not being able to recall something from memory is a lapse. In an error, the action proceeds as planned but fails to achieve its intended outcome because the planned action was wrong, indicating a failure of planning. The situation might have been assessed incorrectly, and/or there could have been a lack of knowledge of the situation. In medicine, slips, lapses, and errors are all serious and can potentially harm patients [2]. In addition, there are many types of medical errors beyond those regarding medication, such as surgical mistakes or skill deficiencies, as well as misdiagnoses [3]. Here, we focused on prescription errors, one subtype of medication errors.

Antimicrobials are the most common incorrectly prescribed drug class [4–6]. The majority (80%) of antibiotic prescribing takes place in the community and injudicious use of antibiotics is a major factor facilitating the emergence of antimicrobial resistance worldwide [7]. Nevertheless, adequate early empirical antimicrobial treatment is relevant and is associated with a significant reduction in all-cause mortality [8]. However, despite its importance, inappropriate initial antimicrobial therapy for septic shock occurs in about 20% of patients and is associated with a fivefold reduction in survival [9].

Determining the role of medication knowledge deficiency as a proximal cause of errors is important in designing error prevention strategies [5]. About 30.8% of prescribing errors were rated clinically significant and were most frequently related to antimicrobial orders, primarily related to incorrect dosing, and medication knowledge deficiency. Bobb et al. found more errors associated with medication knowledge deficiency than errors associated with patient knowledge deficiency [4]. Despite a 50% decrease in preventable adverse drug events with computerized provider order entry [10], this system did not reduce prescription errors and may only be a determinant in pharmacy transcription or validation errors, as well as nursing transcription and dispensation errors [11].

Assessment of clinical competence can be done with different methods. Competence in medicine is defined by Epstein as “the habitual and judicious use of communication, knowledge, technical skills, clinical reasoning, emotions, values and reflection in daily practice for the benefit of the individuals and communities being served” [12] and includes a set of attributes including clinical skills, knowledge, interpersonal skills, problem solving, clinical judgment and technical skills [13]. Competence is contextual, reflecting the relationship between abilities and tasks required to perform in a real-word situation. One of method to evaluate competence in healthcare professionals is the Objective Structured Clinical Examination (OSCE) developed by Harden [14–18]. The reliability and validity of an OSCE increases with the number of stations, although other factors might be involved: having a second rater, nature of items (communication and clinical items), type of rating scale and type of examiner (Standardized patient and content expert) [19].

In the United States, the assessment of medical residents, and increasingly of medical students, is largely based on a model that was developed by the Accreditation Council for Graduate Medical Education (ACGME). This model uses six interrelated domains of competence: medical knowledge, patient care, professionalism, communication and interpersonal skills, practice-based learning and improvement, and systems-based practice. Although accrediting organizations specify broad areas that curriculums should cover and assess, the ideal balance between nationally standardized and school-specific assessment remains to be determined [12]. Some of the components of clinical competence that are assessed by the OSCE include: interrogation, physical examination, laboratory and imaging tests interpretation, diagnosis and management plan, and doctor-patient communication [20]. The relation between ACGME domains and items in our OSCE is: 1) medical knowledge ~ global assessment of knowledge and skills, 2) patient care—physical examination, diagnosis, management plan, and prescription, 3) professionalism ~ communication, and patient’s assessment, 4) communication and interpersonal skills ~ interrogation, communication, and patient’s assessment, 5) practice-based learning and improvement ~ not assessed, 6) systems based practice ~ laboratory and imaging, and management plan.

The OSCE is used to evaluate medical competence at the Faculty of Medicine of the National Autonomous University of Mexico (UNAM). A minimum of 10 stations, which the student usually visits over the course of 3 to 4 h, is necessary to achieve a reliability of 0.85 to 0.90. Previous OSCEs applies of UNAM in seven cohorts achieved a reliability ranged between 0.81 and 0.93 [20]. Under these conditions, structured assessments with the use of standardized patients are as reliable as ratings of directly observed encounters with real patients and take about the same amount of time [12].

Here, we characterize the relationship between clinical competence and antibiotic prescription errors in first-year medical residents in the settings of an OSCE. We also measured the frequency and severity of antibiotics prescription errors and identified items and attributes of clinical competence which were associated with antibiotics prescription error ratio.

Our hypothesis was that the antibiotic prescription errors are related with clinical competence because knowledge is a key component of clinical competence. As part of a construct, knowledge may be related with other components of clinical competence. Our research questions were 1) What is the relationship between clinical competence and antibiotic prescription errors? 2) If clinical competence and antibiotic prescription errors are related, to what extent does clinical competence has influence in antibiotic prescription errors? 3) Which items of clinical competence are related with antibiotic prescription errors? 4) Do these items have an underlying factor that we can estimate indirectly? And 5) How can we measure antibiotic prescription errors with the current tools of assessment in medical education?

## Methods

### Study design, participants, and settings

We designed a cross-sectional study using an OSCE and applied it to first-year medical residents at three medical institutions in Mexico City in February 2019, before the beginning of the specialty courses.

Medical institutions selected were “Manuel Gea Gonzalez” General Hospital, “Salvador Zubirán” National Institute of Medical Sciences and Nutrition and the National Institute of Pediatrics.

We invited to participate first year medical residents from the selected institutions belonging to a direct-entry medical specialty (General Surgery, Gynecology, Internal Medicine or Pediatrics). Residents who had ≥ 12 h of continuous work were excluded from the study. This study was approved by research and ethics committees of all three institutions: “Manuel Gea Gonzalez” General Hospital (approval no. 39–26-2018), “Salvador Zubirán” National Institute of Medical Sciences and Nutrition (approval no. 2863) and the National Autonomous University of Mexico (UNAM) Faculty of Medicine, (approval no. 021/PECEM/2018). The identity of the residents was anonymized by masking, pseudo-anonymization, and aggregation. All residents signed the informed consent document.

### Objective Structured Clinical Examination (OSCE)

#### Instrument design

We designed an OSCE which included guidelines for the examiner, reported by Martinez-Gonzalez et al. with Cronbach’s alpha = 0.94 [21]. Participants were assessed in nine OSCE stations, which were dynamic, had one rater, one standardized patient with an infectious disease clinical case including: pulmonary tuberculosis, acute pyelonephritis, latent syphilis, community acquired pneumonia, acute pharyngitis, acute gastroenteritis, gonorrheal urethritis, cellulitis and acute cystitis. All infectious disease cases were selected according to their outpatient prevalence and the complexity of clinical case was targeted to the knowledge level of a general physician. Each case and its related treatment were approved by independent consensus of two Infectious Disease physicians in active clinical practice, members of the Mexican association for Infectious Diseases and Clinical Microbiology. We took into consideration both the local antibiotic resistance patterns as well as the suggested empirical treatments as per national and international guidelines: pulmonary tuberculosis [22–25], acute pyelonephritis [26–28], latent syphilis [29–31], community acquired pneumonia [32–34], acute pharyngitis [35–40], acute gastroenteritis [41–43], gonorrheal urethritis [44–48], cellulitis [49, 50] and acute cystitis [26, 27, 51, 52].

In addition, solutions to all clinical cases regarding diagnosis were consistent with a medical diagnostic decision support system (DXplain™) [53, 54]; Correct answers regarding treatment were determined by standards of care of the most likely diagnosis in each case. All stations were tested and improved with family medicine residents.

Guidelines and rating scales [21] for each clinical competence component included anamnesis, physical examination, laboratory and imaging tests, diagnosis, therapeutic plan, communication, and patient’s assessment. Components were evaluated as follows:

- Therapeutic plan included pharmacologic and non-pharmacologic actions to manage the patient’s disease from each station including vaccination, hygienic measures, dietary measures, changes in lifestyle, pain and symptom management.
- Assessment by the patient in each station is important to assess interpersonal skills. This was given from the patient by answering the question “How does the physician makes the patient feel?” and ranged from distrust, apathy, coldness, mistreatment to trust, empathy, attention and kindness.
- Prescription and global assessment of knowledge and skills items were measured with rating scales at every station.
- The global assessment of knowledge and skills item was measured by the rater criterion. The rater had specific guidelines for each of the stations and rating considers the overall performance of the resident. The rater can assess the knowledge of the resident when the resident explains the disease to the patient, whilst evaluating the procedures, and medical treatment.
- The prescription item allowed the rater to assess the quality and accuracy of the medical prescription document, written by the junior medical resident, and range from wrong drug to right drug in all “5 rights” (right patient, right drug, right dose, right route, and right time) using the information that the resident wrote [55]. Each OSCE stations has one or multiple solutions and they range totally erroneous solutions to perfect correct solutions

Antibiotic prescription errors ratio was obtained with the number of antibiotics errors in OSCE / “5 rights” per 9 stations. The rate of prescription errors was calculated as the sum of each type of error in a single prescription per evaluation station. Overall, the maximum number of errors in a single prescription was 5.

Severity of prescription errors was measured by the author of all stations, with the National Coordinating Council for Medication Errors Reporting and Prevention (NCC MERP) index, which has acceptable validity and reliability: Agreement = 67.9- 74.6%, 74% accuracy against gold standard of actual harm agreed by panel of experts [56].

#### Instrument raters

A total of eighteen volunteer raters from the Faculty of Medicine at UNAM took part in the assessment process. All raters were physicians and had taken an OSCE workshop of 15 h where they developed one OSCE station. Raters reviewed the stations and guidelines for the examiner before the test. In addition, they were updated with antibiotic treatments of stations. Each rater used an op-scan sheet to score each student with the rating scale. All raters had experience with OSCE (14 raters with 10 years of experience and 4 raters with 3 years of experience) and previously participated in 3–4 OSCEs each year.

#### Standardized patients

A total of eighteen standardized patients participated in the examination. All standardized patients took an OSCE workshop course of 4 h and an acting course of 4 h. They had similar age according to clinical cases. Moreover, they were provided with a dialogue guideline that includes patient personality. All standardized patients had experience with OSCE (6 patients with 10 years of experience and 12 patients with 3 years of experience). In addition, they participated in 3–4 OSCEs each year.

#### Bias management

Most observational studies of prescription errors have found no difference when adjusting the results by the academic level of the physicians, environmental factors at the time of prescription, or the difficulty and type of clinical cases. This OSCE was designed to simultaneously assess the clinical competence and prescription errors in junior medical residents, adjusting by time elapsed since graduation. Furthermore, we considered the local antibiotic resistance of etiologic agents, avoided including fatigued physicians and restricted the access to medical databases during the assessment process.

Following the results of other OSCEs [17, 20] which detected sources of errors through G theory, we designed this OSCE to achieve minimal sources of errors by including trained examiners and standardized patients. The source of errors can be detected with the variance of each variable (in OSCE: examiners, versions, day of application, and stations) that could contribute with the observed score of a person [17]. The assessment was applied in 2 different days depending on the institution in the same schedule.

### Statistical analysis

#### OSCE Rating and assessment

Guidelines for examiners had a rating scale for each clinical competence component: 0.25, insufficient; 0.5, adequate; 0.75, good and 1, excellent. Weighting of the clinical competence score was anamnesis, 30%; physical examination, 12%; laboratory and imaging testing, 16%; diagnosis, 12%; therapeutic plan, 12%; communication, 12% and patient, 6%. Global assessment of knowledge and prescription skills did not contribute to the overall clinical competence score.

Internal consistency of the instrument, both inter-station and inter-item, was measured with Cronbach’s alpha. Cronbach’s alpha provide a measure of the internal consistency of a test or scale. It is expressed as a number between 0 and 1. Internal consistency describes the extent to which all items in a test measure the same concept or construct. Acceptable values of alpha range from 0.70 to 0.95 [57].

Generalizability was measured using the G coefficient. The G coefficient measures the proportion of the total variation produced by the variation in knowledge and skills of the students. A higher value of G implies that the other sources of variation are less important compared to the variation among students. In previous OSCEs the G coefficient was 0.51 to 0.78. We used the estimated variance components for each of the following facts: stations, day of the assessment, medical resident and all their interactions [17].

#### Relationship between clinical competence and antibiotics prescription error

Variable distribution proportions were assessed using Anderson–Darling, D'Agostino & Pearson, and Shapiro–Wilk normality tests [53, 54]. To describe the relationship between clinical competence and antibiotic prescription error ratio we used Pearson´s correlation coefficient and simple linear regression. Spearman´s correction for attenuation was applied to assess the relationship between clinical competence and antibiotic prescription error ratio [58]. An exploratory factor analysis (EFA) was applied to describe factors of clinical competence which were correlated with antibiotic prescription errors, using maximum likelihood extraction and Equamax rotation method [59].

Data analysis was conducted using IBM SPSS 25, R and JMP 11 SAS software.

#### Sample size

We performed sample size estimation prior to study recruitment. A sample size of 49 medical residents was estimated to be sufficient to show correlation between the main variables with *r* = 0.39, α = 0.05 and 80% of statistical power [60]. The sample size consisted of 51 medical residents.

## Results

### Study participants

The number of eligible participants was ~ 255. It was approximated with the meeting attendance of different hospitals. OSCE was applied to 51 (~ 20%) medical residents in February 2019, with a female predominance (60.8%). Most residents were admitted to a pediatrics residency, followed by medical genetics with a median of 7 months of graduation from medical school (IQR 5–18). Most medical residents were from National Institute of Pediatrics (Table 1).

**Table 1:** Participating junior residents characteristics (*n* = 51)

*Characteristic*	*Value (%), Mean* ± *SD (min–max) or Median (Q*_*1*_*-Q*_*3*_*)*
Female	31 (60.8%)
Nacional Institute of Pediatrics	44 (86.3%)
“Manuel Gea González” General Hospital	7 (13.7%)
Pediatrics	41 (80.4%)
Medical genetics	3 (5.9%)
Internal medicine	2 (3.9%)
Traumatology and orthopedics	5 (9.8%)
ENARM^a^ score	78.082 ± 3.03 (71.7—84)
ENARM^a^ 1 time	31 (60.8%)
ENARM^a^ 2 times	12 (23.5%)
ENARM^a^ 3 times	8 (15.7%)
Months after graduation from medical school	7 (5–18)

^a^National Test for Aspirants to Medical Residency (ENARM)

### OSCE results and reliability

**Fig. 1:**
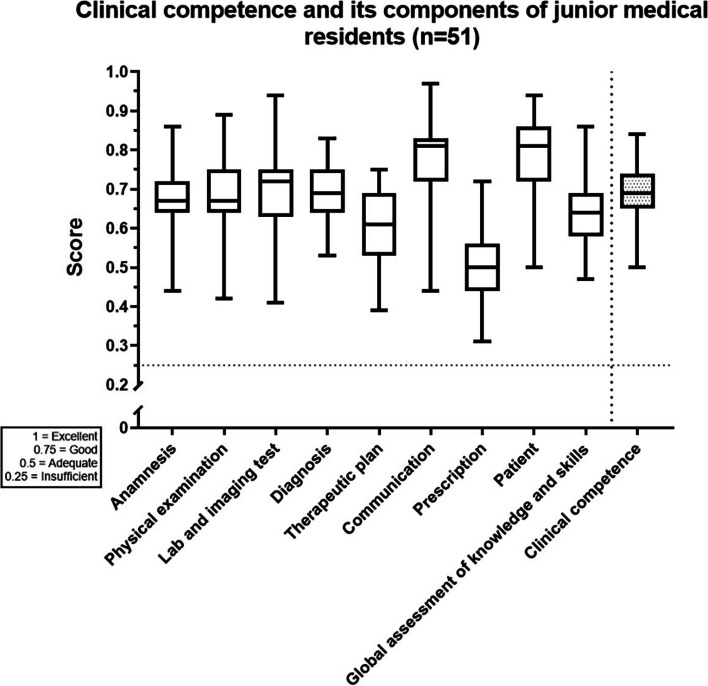
Clinical competence and its components in junior residents (*n* = 51) in an infectious disease OSCE. The score scale is 1 = Excellent, 0.75 = Good, 0.5 = Adequate and 0.25 = Insufficient

Mean OSCE score was 0.692 ± 0.073 SD (Fig. 1). Disaggregated mean clinical competence component scores are: anamnesis 0.682 ± 0.091, physical examination 0.686 ± 0.093, lab and imaging test 0.693 ± 0.099, diagnosis 0.686 ± 0.079, therapeutic plan 0.604 ± 0.097, communication 0.778 ± 0.099 and patient 0.781 ± 0.098. In addition, we showed additionally measured items: prescription 0.496 ± 0.080 and Global assessment of knowledge and skills 0.644 ± 0.082. The OSCE score had a normal distribution. The inter-item Cronbach’s alpha was 0.927 and inter station Cronbach’s alpha was 0.774. The G coefficient from the generalizability analysis was 0.84.

### Antibiotic prescription errors

**Fig. 2:**
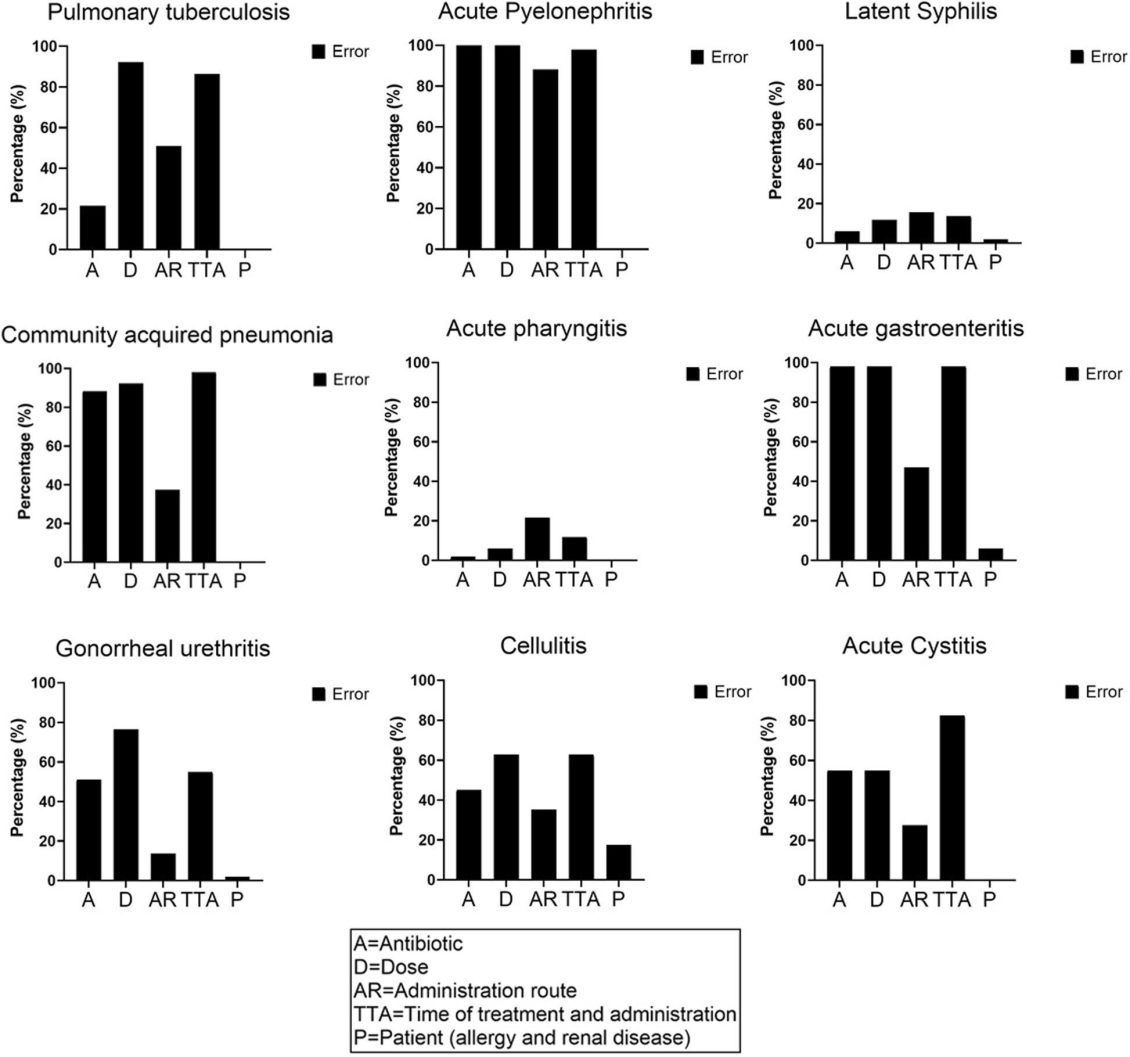
Antibiotic prescription errors ratio using “the 5 rights” (antibiotic, dose, administration route, time of treatment and administration and patient) in each OSCE station

The antibiotic prescription error ratio was 0.451 ± 0.07 SD. Higher rates of prescription errors were observed regarding dosage, administration route and time. The antibiotic prescription error ratio for each station is depicted in Fig. 2. The median rate of prescription errors from each station was: pulmonary tuberculosis 3/5, acute pyelonephritis 4/5, latent syphilis 0/5, community acquired pneumonia 3/5, acute pharyngitis 0/5, acute gastroenteritis 3/5, gonorrheal urethritis 2/5, cellulitis 3/5, and acute cystitis 3/5.

**Fig. 3:**
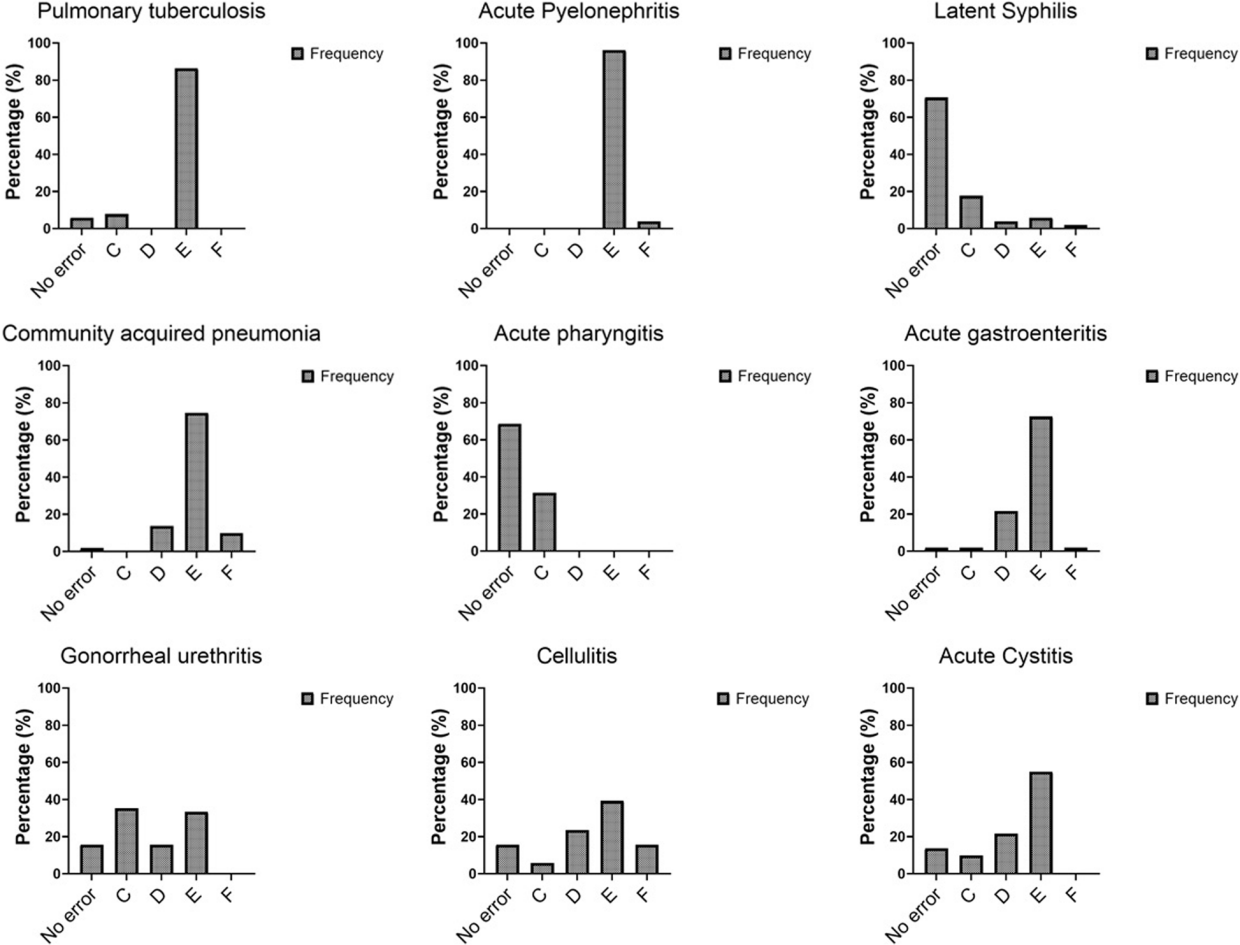
Antibiotic prescription errors severity ratio (NCC MERP Index) in each OSCE station

The severity of antibiotic prescription errors were: category C (errors that do not cause patient harm) in 56 cases (15.5%); category D (monitoring required to confirm that errors resulted in no harm to the patient or intervention required to preclude harm) in 51 cases (14.1%); category E (errors that may contribute to or result in temporary harm to the patient and require intervention) in 235 (65.2%); category F (errors that may contribute to or resulted in temporary harm to the patient and require initial or prolonged hospitalization) in 18 cases (5%). Categories A, B, G, H and I from the NCC MERP index [56] were not considered in this study. The severity of prescription errors at each station is shown in Fig. 3.

### Association between clinical competence and prescription errors

We observed an association between the clinical competence and antibiotic prescription errors ($\rho$=-0.33, 95% CI -0.57 to -0.07, *p* < 0.05), which remained significant after controlling for the effect of gender and time elapsed since graduation from medical school ($\rho$=-0.39, 95% CI -0.625 to -0.118, *p* < 0.01). The correlation increases to $\rho$=-0.68 with the Spearman correction for attenuation [58]. Two outliers were detected and excluded, with a 4.07 and 2.81 anomaly index.

Clinical competence components which showed significant correlations with the prescription antibiotic errors ratio were the therapeutic plan ($\rho$=-0.454, 95% CI -0.713 to -0.135, *p* < 0.05) and the patient’s assessment ($\rho$=-0.351, 95% CI -0.585 to -0.097, *p* < 0.05). The prescription ($\rho$=-0.627, 95% CI -0.771 to -0.46, *p* < 0.05) and the global assessment of knowledge and skills items ($\rho$=-0.45, 95% CI -0.68 to -0.192, *p* < 0.05) also showed significant correlations with prescription antibiotic errors ratio.

**Fig. 4:**
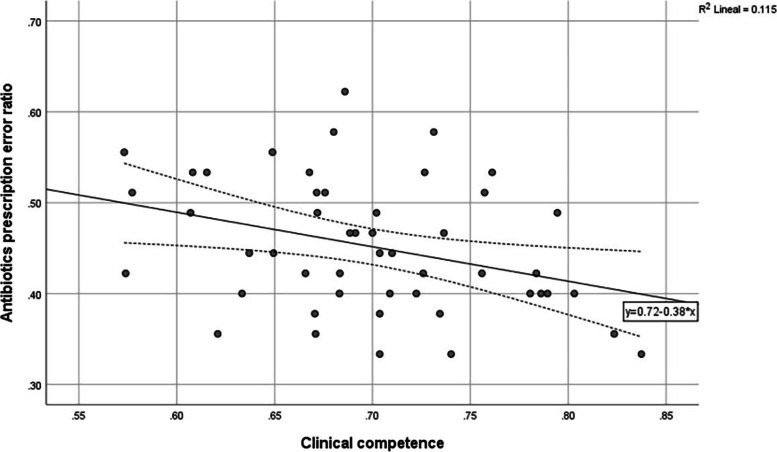
Scatter plot of antibiotic prescription errors ratio and clinical competence. The relationship between the clinical competence and the antibiotic prescription errors is linear (*n* = 49, ${\beta }_{0}$=-17.085, ${\beta }_{1}$=-0.33, *p* < 0.05, CI95% = -31.005, -3.16, ${R}^{2}$=0.11, Residual 0 ± 0.99). The regression line was fitted with least square regression

To know if the clinical competence was useful to predict the antibiotic prescription errors, we used a linear regression (${\beta }_{0}$=-17.085, ${\beta }_{1}$=-0.33, *p* < 0.05, 95% CI = -31.005 to -3.16, ${R}^{2}$= 0.11, Fig. 4), which remained significant after controlling for the effect of gender and time elapsed since graduation from medical school (${\beta }_{0}$=-0.469, ${\beta }_{1}$=-0.418, *p* < 0.01, 95% CI = -0.723 to -0.19, ${R}^{2}$= 0.11).

### Exploratory factorial analysis

We performed an exploratory factor analysis (EFA) to describe additional unobserved variables of clinical competence with the modified OSCE which correlate with the antibiotic prescription errors ratio. Two factors explained 69% of variance using maximum likelihood extraction method.

**Fig. 5:**
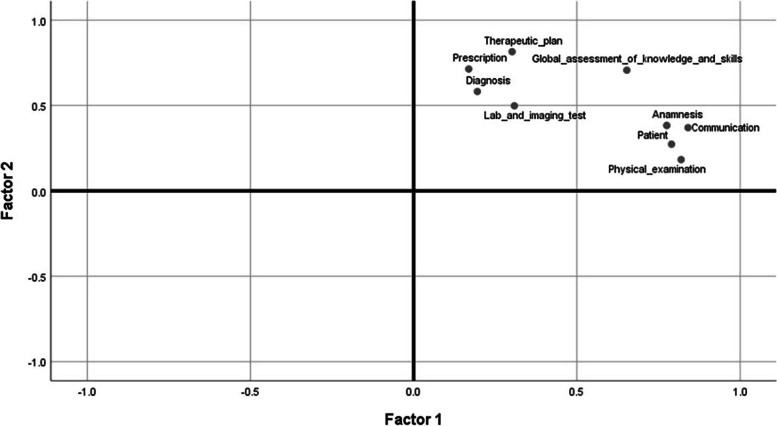
Factor plot in rotated factor space of clinical competence components and OSCE items. Factor 1 (socio-clinical skills) is a cluster by anamnesis, physical examination, communication and patient variables and Factor 2 (diagnostic-therapeutic skills) is a cluster by therapeutic plan, prescription, diagnosis and lab and imaging test variables. Varimax extraction method and equamax method

Factor 1 comprises anamnesis, physical examination, communication and patient and for factor 2 include therapeutic plan, prescription, diagnosis, and laboratory and imaging tests. We labeled factor 1 socio-clinical skills and factor 2 diagnostic-therapeutic skills. The oblique rotation was considered, although the orthogonal rotation using Equamax achieved a simple structure and easier interpretation, as shown in Fig. 5.

Figure 5 shows a factor plot in rotated factor space with the Equamax method. The x-axis shows the loadings for factor 1 and the y-axis shows the loadings for factor 2 Factor loading is basically the correlation coefficient (dimensionless) for the variable and factor. Factor loading shows the variance explained by the variable on that factor. The items are organized in the common factor space and shows its contributions to each factor, global assessment of knowledge and skills is the item that contributes to the 2 factors and is a complex variable. Therapeutic plan item has the highest contribution to factor 1 and communication to factor 2.

Factor 2, diagnostic-therapeutic skills was correlated with antibiotic prescription error ratio (*r* = -0.536, 95% CI -9.24 to -3.43, *p* < 0.001). To know if the factor 2 was useful to predict the antibiotic prescription errors, we used a multiple linear regression controlling for the effect of gender and time elapsed since graduation from medical school (${\beta }_{0}$=-0.047, ${\beta }_{1}$=-0.552, *p* < 0.01, 95% CI = -0.064 to -0.027, ${R}^{2}$= 0.27).

## Discussion

Our results show a negative correlation between clinical competence and the antibiotic prescription error ratio. The strength of association increased when we corrected the attenuation and considered sex and months since the physician’s graduation from medical school. This study characterizes the influence of clinical competence on the antibiotic prescription of junior physicians. In addition, it shows the role of the attributes of clinical competence in antibiotic prescription errors. Our results give measures to some points that were explored in qualitative research in United Kingdom, which developed an educational tool which delivered knowledge in (i) principles of antimicrobial prescribing, (ii) diagnosis of infections, (iii) clinical review of patients with infections, (iv) prescribing in the context of antimicrobial resistance, and (v) the role of laboratory testing and test results in prescribing [61]. Previous studies describe that dosage calculation is a particular cognitive challenge, which had the higher error in all stations in our study [62]. Our study is the first to demonstrate that the clinical competence, assessed with an OSCE, is predictive of antibiotic prescription errors.

The correlation between clinical competence and errors in antibiotic prescription increases to *ρ* = -0.68 with the Spearman correction for attenuation compared to Pearson’s correlation coefficient. Spearman’s correction for attenuation has proven useful over the years primarily because it allows researchers to determine what the linear relationship would be between two variables, X and Y, if all measurement errors were removed from one or both variables. Spearman’s correction uses the internal consistency of the items as the divisor. Then, Spearman’s correction will increase if the internal consistency is low. The correction is especially useful for looking at the strength of theoretical relationships between variables undistorted by measurement errors [58]. However, we preferred focus on original value of Pearson’s correlation because the internal consistency of frequency of prescription errors is poor. The reason may be that the frequency of antibiotic prescription errors is case specific, which means that antibiotic prescription errors on one clinical case did not necessarily predict antibiotic prescription errors in another [63].

The implemented OSCE is reliable because its internal consistency measured with Cronbach’s alpha and generalizability coefficient is higher than reported values of the systematic review of the literature [17, 19].

Despite the fact that this OSCE is specifically aimed to evaluate prescriptions in settings of infectious diseases consultation, our score is similar to a study in 7 generations of physicians, just at the end of their medical degree [20]. Moreover, similar scores were reported in Switzerland and the United States of America [64, 65].

Interestingly, the antibiotic prescription error ratio in this study is higher than the previous international reports [4, 5, 11, 66, 67], but similar to national estimates [6, 68]. In a prospective cohort study, the antibiotics prescription error ratio was 18% in patients hospitalized in internal medicine wards. Moreover, patients with adverse drug events (ADEs) had more antibiotics prescriptions errors; the most common infectious diseases were urinary tract infection and pneumonia [66]. We included recommended diagnoses in Delphi´s consensus from the Netherlands [69], urinary tract infections and pneumonia in our OSCE.

The antibiotic and dose errors in acute pyelonephritis and gastroenteritis are very high. We noted that national guidelines were outdated and did not consider the current antimicrobial resistance rates and current recommendations regarding first-line treatment for pyelonephritis. The only treatment available to acute pyelonephritis in outpatient settings is ertapenem because E. coli has ≥ 34% ciprofloxacin resistance and > 30% ceftriaxone resistance in Mexico. The problem is similar with gastroenteritis, where the only treatment available was azithromycin. Gharbi et al. proposed the topic “prescribing in the context of antimicrobial resistance” in the intervention to optimize prescribing practice. Omitting antimicrobial resistance is a deficiency in the medical curriculum. However, the use of outdated national guidelines in a country and the quick increase of antimicrobial resistance may be outside the scope of medical education. Furthermore, getting updated information could be complicated in the clinical environment, especially with the patient. This is an area of opportunity to improve training in the prescription of antimicrobials in Mexico to promote evidence-based prescription in an outpatient setting.

One of the objectives of the study was to find items and attributes of clinical competence that are associated with antibiotic prescription error ratio. The therapeutic plan, which is a component of clinical competence, showed a negative correlation with antibiotic prescription errors. In addition, we introduced a prescription item in synthetic guidelines for the rater in the OSCE to assess the medical prescription making process; this prescription item showed a strong negative correlation with antibiotic prescription errors and high internal consistency with other items. We translated the frequency of errors (“5 rights” of each prescription) into an item. Then, it allowed assessment by the OSCE rater using a rating scale. The correlation with the frequency of prescription errors is logic and is a proof of the good assessment of raters. In the future, the prescription item could be included in the components of clinical competence score to get a better assessment.

The current clinical competence score only explains 11% of antibiotic prescription errors ratio in the linear model; indicating potentially unobserved phenomena which additionally contribute to prescription error and which should be evaluated in the future studies, particularly the complex interaction of health care culture with antimicrobial prescription [70].

Many factors influence prescription errors: lack of knowledge regarding drug therapy, lack of knowledge about patient factors that affect drug therapy, calculations and terminology [5, 71]. They are preventable and are ranked according to their frequency [4, 72]. We measured the medication errors at the prescribing stage in antibiotics because of its important implications and the high frequency of errors in this group. The OSCE measured the application of knowledge in a task, and the items regarding prescription and therapeutic plans took into consideration the common factors reported in medication errors. Of note, most of the antibiotic prescription errors severity fell in category E (65.2%), indicating a high severity and a necessity of intervention in patient care. A second therapeutic intervention in patients with an infectious disease has many clinical consequences, it increases the cost, leads to antibiotic resistance and increases mortality [8, 73, 74]. The impact of these errors in patient´s outcomes and the mediating effect of clinical competence in moderating this impact should be a focus for future studies and could indicate potential areas for intervention within medical education curriculum.

Our results support an association between clinical competence and antibiotic prescription error ratio. Notably, the diagnosis item was not correlated with antibiotic prescription errors, but only the therapeutic plan. A possible explanation is that most errors occur along the therapeutic reasoning process.

All components of clinical competence have relation and sequential logic. Nevertheless, we identified that these items primarily clustered into two factors. Exploratory factorial analysis shows two underlying factors of the items. Diagnostic-therapeutic skills have a moderate correlation with antibiotic prescription errors. Socio-clinical skills were not related. Thus, the correlation emphasized the relevance of this underlying factor within the construct of clinical competence.

The contributions of this research to medical education are four-fold: First, we showed the possible factors underlying the OSCE´s items and most importantly, the relationship between medical competence and antibiotic prescription errors. Second, we characterized the influence of clinical competence in antibiotic prescription errors and suggesting areas of opportunity for future research in other factors that have influence in antibiotic prescription errors (eg. usual prescriptions in local healthcare institutions, searching information skills with devices in healthcare environment and the standard answers of exams to enter in a medical specialty course and slips or lapses of deficit in attention). Third, we proposed a method to quantify antibiotic prescription errors in a controlled environment which is safe to the patients and physicians using a reliable OSCE which is specific for infectious diseases. Four, we show the potential relevance of including the human error and patient safety in the medical as well as a practical training process to improve antimicrobials prescription.

If we want to improve medical education to reduce prescription errors, we need to continue this research field. We suggest assessing different interventions in the clinical competence and quantifying the antibiotic prescription errors. Future studies may include simulation training to increase clinical competence and to give feedback. In addition, interventions should include prescribing in the context of antimicrobial resistance.

### Limitations

Medical residents voluntarily participated in this study, and we did not assess medical residents who refused the test, which could introduce bias in the results if clinical competence might have factored into this decision. We consider that the voluntary participation lead a minimum use of the simulation [75, 76] and the effect of motivation remains unknown; however, the time of the OSCE, residents did not have access barriers and the reason may be the perception of OSCE. Moreover, residents selected these institutions previously with their own selection process and clinical competence might be skewed in this process compared to the general population of junior residents in Mexico. Furthermore, overrepresentation of pediatrics residents might reduce representativeness for other medical specialties. For these reasons, the score may not be a representative measure of the clinical competence across all general physicians.

In addition, slips, lapses and mistakes were considered as the same error in this study because our tools and resources did not allow discriminating them. The author of OSCE stations (MD, cPhD) and two specialists in Infectious Diseases agreed on severity of antibiotic prescription errors using the National Coordinating Council for Medication Errors Reporting and Prevention (NCC MERP) index, but it was not independent agreement. We did not measure inter interrater reliability.

Another potential source of bias is medical residents previous experience with OSCE. The effect on scores from medical residents with previous experience with OSCE remains unknown, although the evidence shows that it does not modify subsequent performance scores [77]. Previous experience is highly dependent on the university of origin and its assessment methods; however, OSCE exposure and subsequent performance has shown a weak correlation [78]. Additionally, the OSCE is a simulation and may be inflexible, and its performance in superior years of specialty course is unknown.

## Conclusions

We observed a negative correlation between clinical competence and antibiotic prescription error ratio in junior medical residents who have been accepted to a medical specialty. The therapeutic plan, which is a component of clinical competence score, and the prescription skills had a negative correlation with antibiotic prescription errors. The most frequent mistakes in antibiotic prescriptions errors would require a second intervention.

Our findings contribute to the evolving understanding of medication errors in the prescription stage of antibiotics. Assessing is a part of medical curriculum and medical education. We developed a method to measure the antibiotic prescription errors without risk to the patients. This study adds important evidence to improve the curriculum and medical education to avoid antibiotic prescription errors, thus increasing patient safety and reducing costs, mortality and antibiotic resistance.

## Supplementary Information

**Additional file 1:** **Supplementary figure 1.** Plot of the number of methods that suggest the number of factors to retain. The choice of 2 dimensions is supported by 8 (34.78%) methods. **Supplementary figure 2.** Scree plot that shows the eigen values of factors and components.

## Data Availability

All data (data base in.sav format)) and materials (guidelines for the examiner and rating scales) are available under reasonable request by writing to the email adrianmartinez38@gmail.com.
