# Supplementary material for: Antibiotic prescription errors: the relationship with clinical competence in junior medical residents
Source: BMC Med Educ. 2022 Jun 14;22:456. doi: 10.1186/s12909-022-03499-0 (PMC9199232; doi:10.1186/s12909-022-03499-0)
Supplement: Supplementary file 1 — Additional file 1: Supplementary figure 1. Plot of the number of methods that suggest the number of factors to retain. The choice of 2 dimensions is supported by 8 (34.78%) methods. Supplementary figure 2. Scree plot that shows the eigen values of factors and components. [file 12909_2022_3499_MOESM1_ESM.docx]

Supplementary figure 1. Plot of the number of methods that suggest the number of factors to retain. The choice of 2 dimensions is supported by 8 (34.78%) methods.


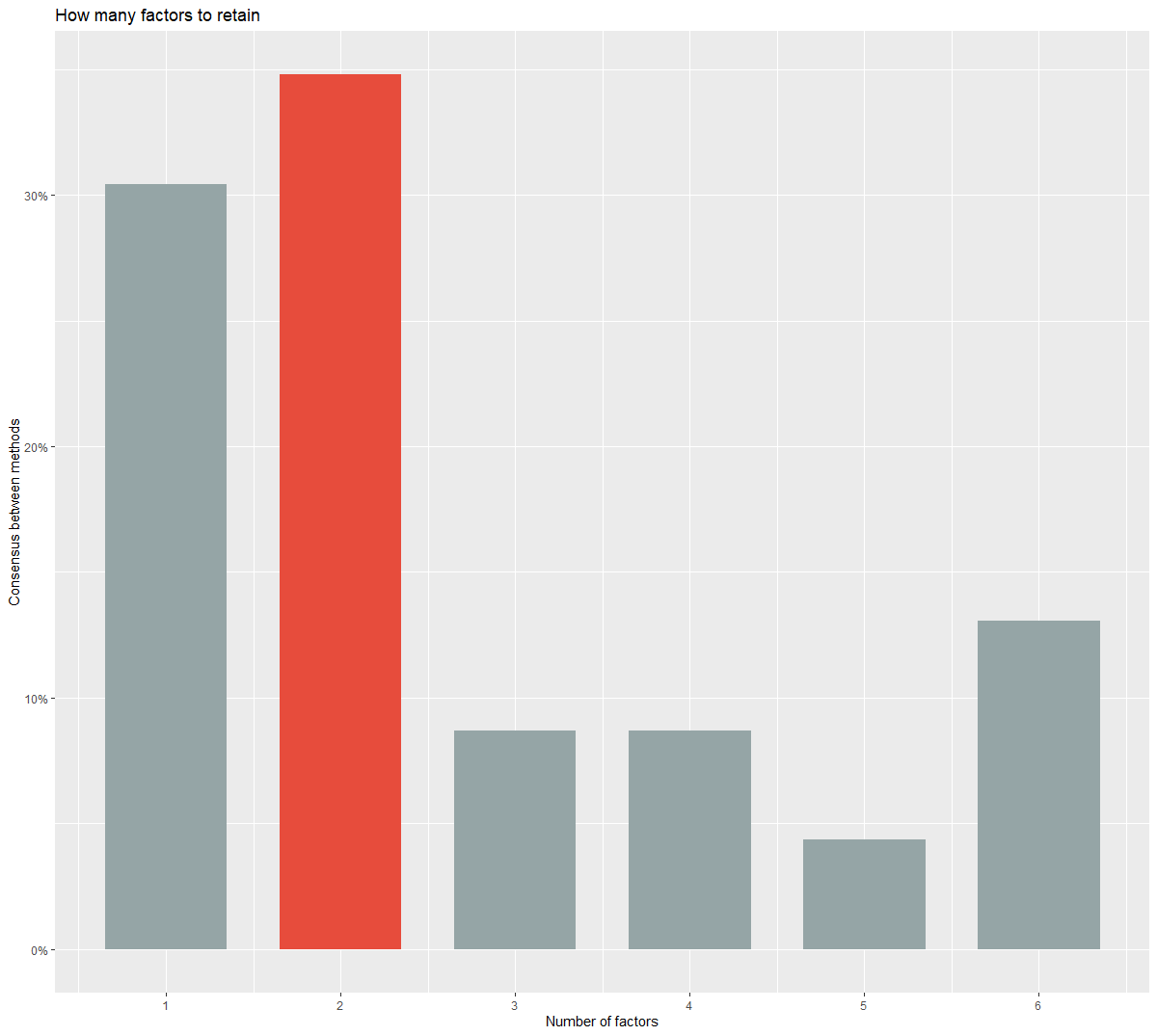


The choice of 2 dimensions is supported by 8 (34.78%) methods out of 23 (Optimal coordinates, Parallel analysis, Kaiser criterion, SE Scree, VSS complexity 2, Velicer's MAP, BIC, BIC).

n_Factors Method Family

1 1 t Multiple_regression

2 1 p Multiple_regression

3 1 Acceleration factor Scree

4 1 R2 Scree_SE

5 1 VSS complexity 1 VSS

6 1 TLI Fit

7 1 RMSEA Fit

8 2 Optimal coordinates Scree

9 2 Parallel analysis Scree

10 2 Kaiser criterion Scree

11 2 SE Scree Scree_SE

12 2 VSS complexity 2 VSS

13 2 Velicer's MAP Velicers_MAP

14 2 BIC BIC

15 2 BIC Fit

16 3 Bentler Bentler

17 3 CNG CNG

18 4 beta Multiple_regression

19 4 BIC (adjusted) BIC

20 5 CRMS Fit

21 6 Bartlett Barlett

22 6 Anderson Barlett

23 6 Lawley Barlett

Supplementary figure 2. Scree plot that shows the eigen values of factors and components.


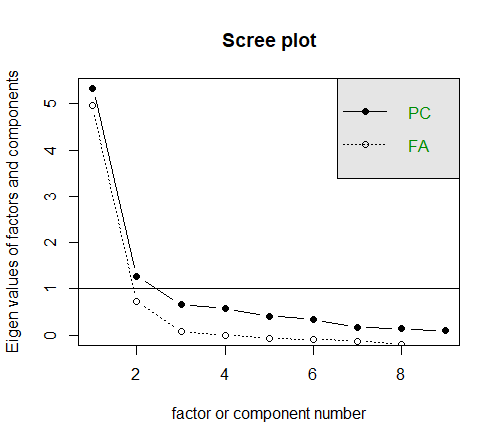


Factor Analysis using method = ml

Call: fa(r = resi, nfactors = 2, rotate = "equamax", fm = "ml",

cor = "mixed")

Standardized loadings (pattern matrix) based upon correlation matrix

item ML1 ML2 h2 u2 com

Communication 6 0.86 0.32 0.84 0.157 1.3

Physical_examination 2 0.83 0.13 0.70 0.296 1.1

Patient 8 0.80 0.22 0.70 0.302 1.2

Anamnesis 1 0.80 0.33 0.75 0.253 1.3

Global_assessment_of_knowledge_and_skills 9 0.69 0.67 0.93 0.075 2.0

Therapeutic_plan 5 0.35 0.79 0.75 0.245 1.4

Prescription 7 0.21 0.70 0.54 0.462 1.2

Diagnosis 4 0.23 0.57 0.38 0.624 1.3

Lab_and_imaging_test 3 0.34 0.48 0.34 0.657 1.8

ML1 ML2

SS loadings 3.53 2.40

Proportion Var 0.39 0.27

Cumulative Var 0.39 0.66

Proportion Explained 0.60 0.40

Cumulative Proportion 0.60 1.00

Mean item complexity = 1.4

Test of the hypothesis that 2 factors are sufficient.

The degrees of freedom for the null model are 36 and the objective function was 7 with Chi Square of 323.28

The degrees of freedom for the model are 19 and the objective function was 0.43

The root mean square of the residuals (RMSR) is 0.03

The df corrected root mean square of the residuals is 0.04

The harmonic number of observations is 51 with the empirical chi square 3.92 with prob < 1

The total number of observations was 51 with Likelihood Chi Square = 19.37 with prob < 0.43

Tucker Lewis Index of factoring reliability = 0.997

RMSEA index = 0 and the 90 % confidence intervals are 0 0.126

BIC = -55.33

Fit based upon off diagonal values = 1

Measures of factor score adequacy

ML1 ML2

Correlation of (regression) scores with factors 0.94 0.91

Multiple R square of scores with factors 0.89 0.82

Minimum correlation of possible factor scores 0.78 0.65
